# Enhancement of fluorescence and anti-tumor effect of ZnO QDs by La doping

**DOI:** 10.3389/fchem.2022.1042038

**Published:** 2022-10-10

**Authors:** Ruixin Hao, Shanshan Luo, Feiyan Wang, Xinyu Pan, Jing Yao, Jielian Wu, Haihong Fang, Wenkui Li

**Affiliations:** ^1^ Jiangxi Provincial Key Laboratory of Drug Design and Evaluation, Jiangxi Science and Technology Normal University, Nanchang, China; ^2^ Jiangxi Provincial Key Laboratory of Surface Engineering, Jiangxi Science and Technology Normal University, Nanchang, China

**Keywords:** ZnO, quantum dots, fluorescence, ion doping, drug delivery

## Abstract

ZnO quantum dots (QDs) have received much attention as biomarkers and drug delivery systems in cancer treatment, due to their low cost, ease of preparation, and pH-responsive degradation. However, its applications are limited by the low quantum yield and light absorption. In this work, a lanthanum-doped zinc oxide (La-ZnO) QDs-based drug delivery platform was constructed. The results show that 4% La doping is the most beneficial for improving the fluorescent properties of the ZnO QDs. After loading the drug, the cell activity was 15% at ZnO@DOX and 12% at La-ZnO@DOX. According to *in vitro* and *in vivo* experiment results, the La-ZnO QDs show enhancement of the antitumor effect. Dual enhancement of fluorescence and anti-tumor effects make La-ZnO QDs promising as a drug delivery system in cancer treatment.

## 1 Introduction

Nanomaterial composites, combined functions such as imaging, targeting, drug delivery, and cancer treatment, show great potential for applications in anticancer medicine (Liu et al., 2007; Wang et al., 2020; Sahu et al., 2021; Khan et al., 2022). Last decades, quantum dot clusters attracted high interest in bioimaging and drug delivery (Zrazhevskiy et al., 2010; Probst et al., 2013). Quantum dots (QDs) mainly play two roles in the drug transport system. Firstly, QDs can serve as drug carriers to bring drugs to the target. Secondly, QDs can help elucidate the drug metabolism kinetics and pharmacodynamics as fluorescent probes to trace the distribution of drugs *in vivo* (Probst et al., 2013). ZnO is a promising candidate for fluorescent probes due to its low cost, ease of preparation, and pH-responsive degradation (Xiong, 2013; Cai et al., 2016). However, Using ZnO as a biological label in tumor therapy applications is limited by the unstable emission intensity and low quantum yields. The quantum yield of ZnO QDs prepared by the conventional method is usually less than 10% (Gulia and Kakkar, 2013; Verma et al., 2021). How to effectively improve the photoluminescent (PL) properties of ZnO QDs remains to be studied.

Ion-doping is an effective way to improve the electronic, optical, and magnetic properties of semiconductor devices (Thangeeswari et al., 2015; Zheng et al., 2022). As common semiconductor dopants, rare earth (RE) elements have attracted widespread interest because of their unique effect on optical properties (Huang W et al., 2019; Prakash et al., 2021). Zong et al. (2014) prepared Eu-doped ZnO QDs, and the results show that Eu-doped ZnO QDs exhibited better photocatalytic activity than pure ZnO, and they are promising for applications for photodegradation of organic contaminants in wastewater. Cao et al. (2017) prepared Ga-doped ZnO NPs (nanoparticles) and found that charge transfer at the interface of the nanoparticles was attenuated by doped Ga. A few studies have been devoted to the preparation of lanthanum-doped ZnO QDs. Huang P et al. (2019) prepared La-doped ZnO QDs with stable fluorescence performance. Ge et al. (2007) studied the gas sensitivity of La-doped ZnO QDs, and found that proper La doping could enhance the gas-sensitive properties. However, few studies are available on the use of La-doped ZnO QDs as a drug delivery system (DDS) for cancer therapy.

This research aims to develop a ligand/receptor-based targeted drug delivery system using La-ZnO QDs as the core material. Hyaluronic Acid (HA) was used to modify the La-ZnO because HA can specifically bind to the major receptor CD44 (Gomez et al., 2020; Maksoudian et al., 2020; Yusupov et al., 2021). Besides, Polyethylene glycol (PEG) was introduced to stabilize the La-ZnO QDs under physiological conditions (Zhao et al., 2014; Reisch et al., 2015; Cai et al., 2018). Finally, DOX (Adriamycin), an anti-tumor drug, was loaded to HA-La-ZnO-PEG through covalent interactions and the formation of Zn2+-DOX chelating complexes. The results show that the doping of lanthanum achieves a dual enhancement of fluorescence and anti-tumor effect for the drug delivery system. The La-ZnO QDs have good prospects for application in drug delivery systems for cancer therapy.

## 2 Materials and method

### 2.1 Materials

Zinc acetate dihydrate (Zn(CH3COO)2·2H2O) was obtained from Shanghai Titan Co., Ltd. 3-aminopropyl triethoxysilane (APTES) was purchased from Shanghai RHAWN Co., Ltd. Lithium hydroxide (LiOH), N-N-dimethylformamide (DMF), 1-(3-dimethylaminopropyl)-3-Ethyl carbodiimide salt (EDC), N-hydroxysuccinimide (NHS), Hyaluronic acid (HA) were obtained from Aladdin Reagents (Shanghai) Co., Ltd. Adriamycin (DOX) was purchased from Bide Pharmatech Ltd.

### 2.2 Preparation of pure and La-doped ZnO QDs

**FIGURE 1:**
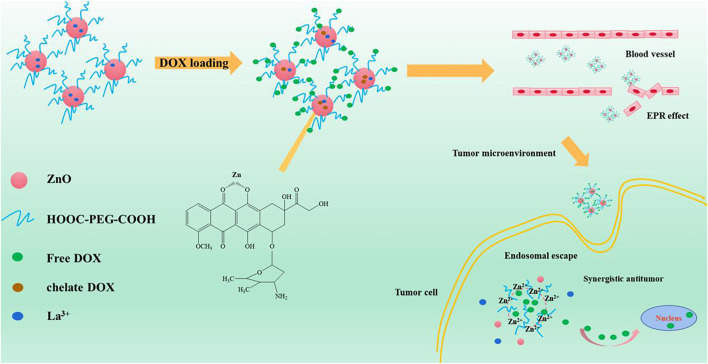
Schematic illustration of the preparation of PEG-HA-La-ZnO and its antitumor effects investigate in both *in vitro* and *in vivo*.

**FIGURE 2:**
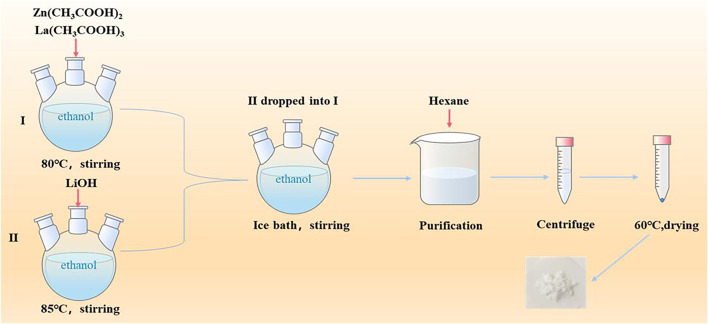
Synthesis process of pure and La doped ZnO QDs.

Figure 1 is the schematic illustration of the preparation of PEG-HA-La-ZnO and its antitumor effects investigate in both *in vitro* and *in vivo*. The steps of synthesizing pure and La-doped ZnO QDs are shown in Figure 2. In the first step, 2.20 g of Zn(CH3COO)2·2H2O and 0%, 2%, 4%, 6%, and 8% lanthanum acetate were dissolved in 100 ml of absolute ethanol solution, then refluxed and stirred for 2 h under 80°C in a water bath until the solution was colorless and transparent. Then the above solution was placed in an ice bath. At the same time, according to the ratio of RZn-OH = 1:1, LiOH was weighed and dissolved in 150 ml of absolute ethanol solution, then heated to 85°C and stirred for 2 h until the solution was colorless and transparent. Then the solution was cooled to room temperature, before adding them to zinc acetate ethanol solution in an ice bath drop by drop for 8 h. It was observed that the zinc acetate solution quickly turned white and then gradually became clear, indicating that ZnO quantum dots had been formed. In the second step, n-hexane was added to precipitate a large amount of white precipitation. The precipitation was then centrifuged for 15 min under the condition of 4500 R/min and finally dried in a vacuum at 60°C to obtain ZnO quantum dots.

### 2.3 Preparation of APTES-modifified La-ZnO QDs

Firstly, 100 mg pure or La-ZnO QDs were dispersed in 15 ml DMF solution under ultrasonic conditions. After heating to 120°C, 50 μL APTES was added and stirred for 15 min, followed by centrifuging at 1,000 rpm for 5 min and washing with DMF twice. Then the amino ZnO QDs were collected and dispersed in 10 ml of water and then stored at 4°C.

### 2.4 Surface modification of La-ZnO QDs with PEG (PEG-La-ZnO)

COOH-PEG-COOH (Mw: 2000) was used to modify the surface of NH2-La-ZnO QDs through amide reaction. At first, 0.2 g EDC/NES was added to 1 ml of ultrapure water containing 25 mg PEG, followed by adjusting the pH value of the mixture to 7 with NaOH. The solution was shaken at 25°C for 30 min to active COOH-PEG-COOH. After activation, 2.5 ml of NH2-ZnO QDs dispersion was slowly added, then the mixture was stirred at room temperature in dark for 4 h. The final product was freeze-dried and preserved after removing the unmodified COOH-PEG-COOH.

### 2.5 Surface modification of PEG-La-ZnO QDs with HA

Firstly, 0.016 mmol of HA was dissolved in 4 ml of ultrapure water, then the pH of the solution was adjusted to four using hydrochloric acid. Then the solution was incubated overnight at room temperature. Afterward, 0.32 mmol EDC/NHS was added to a well-incubated HA solution and activated for 4 h. Finally, 5 mg PEG-La-ZnO QDs were added into the solution and stirred overnight. The obtained HA-PEG-La-ZnO was dialyzed in water with a dialysis bag of molecular weight 3,500 Da for 24 h to remove the excess HA.

### 2.6 DOX loading (PEG-HA-La-ZnO and PEG-HA-ZnO)

The potential of HA-PEG-ZnO and HA-PEG-La-ZnO nanocomposites *in vitro* drug encapsulation was evaluated. Firstly, 10 mg DOX was put in 10 ml ultrapure water to prepare 1 mg/ml of DOX solution. Afterward, 0.32 mmol of EDC and 0.16 mmol of NHS were mixed with HA-PEG-NH2-ZnO solution and stirred for 30 min, followed by adding 1 ml DOX solution. Then the mixture was stirred overnight in dark. Finally, the reaction products were measured by UV/Vis spectroscopy at 490 nm, and the loading content and loading efficiency of DOX were calculated as follows:

$$Loading\;content\;\left(\%\right)=\frac{weight\;of\;DOX\;in\;nanoparticles}{weight\;of\;nanoparticles\;taken}\times 100\%$$ (1)

$$Loading\;efficiency\;\left(\%\right)=\frac{weight\;of\;DOX\;in\;nanoparticles}{weight\;of\;DOX\;added}\times 100\%$$ (2)

#### 2.7 *In vitro* DOX release experiment

PBS buffers (pH = 7.4 and pH = 5.0) were used to simulate the microenvironment of normal cells and cancer cells, respectively. The HA-PEG-La-ZnO DOX sample was put into a dialysis bag with a molecular weight of 3,500 Da and then stirred at the corresponding buffer solution. The fluorescence data were measured at the corresponding time points to obtain the drug release curve.

### 2.8 Cytotoxicity analysis

#### 2.8.1 Cell culture

B16 cells were cultured in a cell culture flask with DMEM, including 10% fetal bovine serum (FBS) and 1% penicillin-streptomycin solution in a humidified atmosphere incubator with 5% CO2 at 37°C.

#### 2.8.2 MTT assay

To explore the biocompatibility of ZnO, B16 cells were used in the experiment. The cells were cultured in a constant temperature incubator at 37°C and the fourth generation was used for the experiment. To assess the toxicity determined by the MTT method, the 1 × 105 cells were put in 96—well plates with different materials (ZnO, La—ZnO)concentrations of 0 ug/ml, 5 ug/ml, 10 ug/ml, 20 ug/ml, 25 ug/ml, respectively. The experiments were repeated three times for every concentration. After 48 h, MTT solution was added. After 4 h, MTT solution was removed and 150 ul DMSO (Dimethyl sulfoxide) solution was added. Finally, the absorbance of B16 cells was measured by a microplate analyzer.

To explore the therapeutic effect of La-ZnO drug loading system, B16 cells were selected as the experimental cells, and the MTT method was used to evaluate the toxicity test. Firstly, 1 × 105 cells and different materials (ZnO @ DOX and 4 -La-ZnO @ DOX) were mixed in 96—well plates for 48 h. The concentration of DOX were 0.162 ug/ml, 0.81 ug/ml, 1.62 ug/ml, 2.43 ug/ml, 3.24 ug/ml, 4.05 ug/ml, respectively. Then added MTT solution for 4 h. Removed MTT solution, and added 150 ul DMSO (Dimethyl sulfoxide) solution to lyse cells. Finally, the absorbance of B16 cells was measured by a microplate analyzer to explore the viability of B16 cells.

#### 2.8.3 Fluorescence staining experiment

Fluorescence staining experiment was used to evaluate the B16 cell activity after different treatments. The cells were cultured with different samples for 2 days. Afterward, the cells was wash twice with PBS, then were stained with AM/PI staining for 30 min, followed by washing with PBS for twice, the cells were photographed by a fluorescence microscope (CKX53, OLYMPUS).

### 2.9 *In vivo* biocompatibility

To evaluate the biocompatibility of ZnO, six tumor-free female C57 mice (4–6 weeks old) were randomly divided into control group and 25 μg/ml ZnO group. Then, bodyweight of the mice in the two groups was recorded with subcutaneous injection of 100 μL of PBS and 25 μg/ml of ZnO solution for 10 consecutive days, and the main organs (liver, heart, spleen, lung, and kidney) were collected for H&E staining.

### 2.10 *In vivo* antitumor activity

Female C57 mice (4–6 weeks old,16–20 g) were used for antitumor activity assessment. Firstly, 100 uL of B16F10 cell suspension (1 × 107) was injected subcutaneously into the lower hind limb of each mouse. After 6–8 days, the tumor volume was about 80 mm3, and the drug was administered. Eighteen tumor-bearing mice were randomly divided into six groups, PBS, ZnO, 4% La-ZnO, DOX, ZnO@DOX, and 4%La-ZnO@DOX, respectively. The equivalent concentrations of ZnO and 4% La-ZnO in PBS were 25 μg/ml, and the equivalent concentrations of DOX, ZnO@DOX and 4%La-ZnO@DOX in PBS were 4.05ug/mL. The tumor size and weight of mice were recorded once a day. After 10 days of recording, the tumors of each group were collected for H&E staining. The tumor volume was calculated by the following equation:

Tumor volume(mm3)=(l×W2)/2 (3)

where *l* is the length, and W is the width in mm of tumor.

### 2.11 Characteristics

The crystal structure and composition were measured through an X-ray powder diffractometer (Shimadzu, Japan, XRD-6100). The morphology and particle size were observed using a field emission transmission electron microscope (FEI Company, United States). Surface functional groups were determined using a Fourier transform infrared spectrometer (IR-960, Tianjin Rui’an Technology Co., Ltd.). The fluorescence performance was tested by a UV spectrophotometer (model UV-2550, Shimadzu, Japan), a PL fluorescence spectroscopy (Beijing ZhuoliHanguang Instrument Co., Ltd.), and a fluorescence spectrometer (Hitachi High-Tech Co., Ltd, Japan).

## 3. Result and discussion

### 3.1 Structure and morphology of ZnO QDs

**FIGURE 3:**
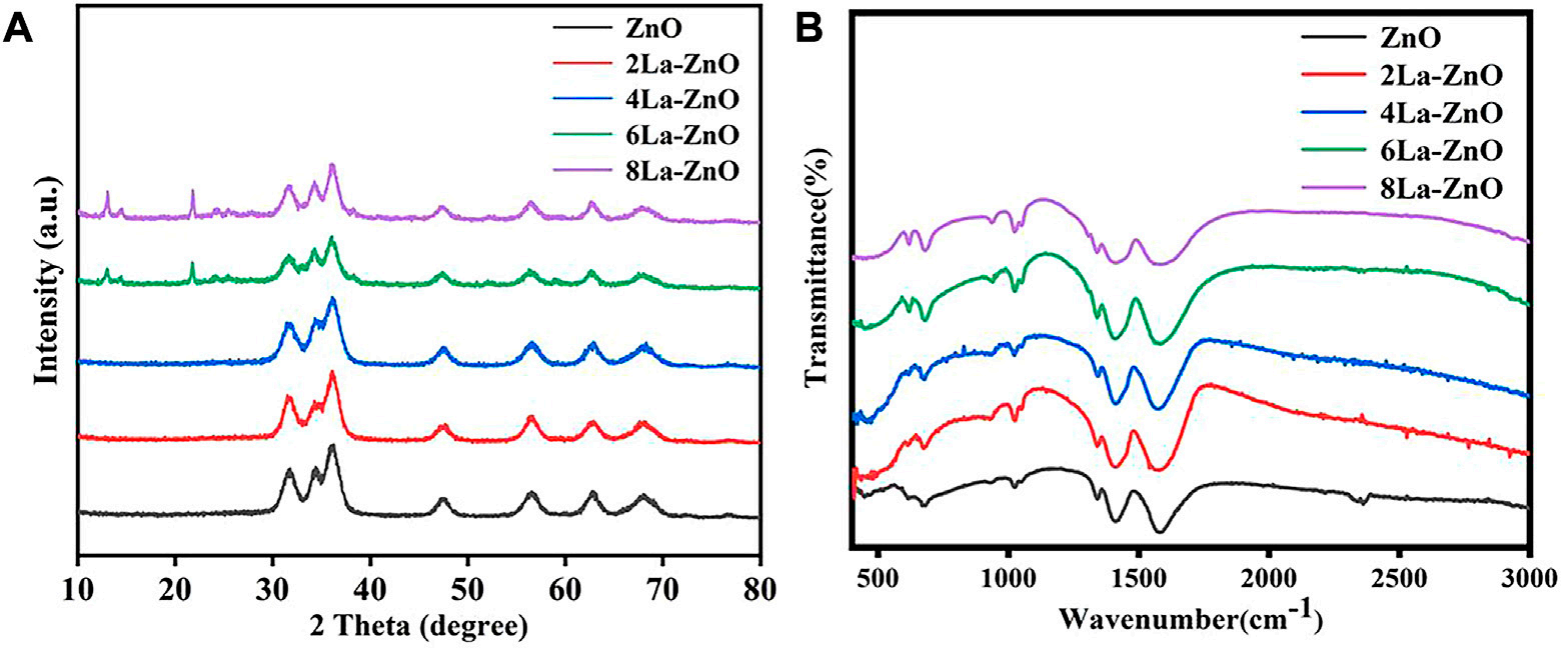
**(A)** XRD pattern and **(B)** FTIR spectra of pure ZnO and La-ZnO QDs.

The XRD patterns of ZnO QDs were exhibited in Figure 3A. The diffraction peaks of the pure ZnO and La-ZnO samples and their relative intensities matched well with the standard JCPDS card No. 36–1,451, and both pure ZnO and La-ZnO QDs in this work were hexagonal wurtzite structures. In addition, no other impurities XRD peaks were observed when the doping La is not exceed 4%, which indicated that the doping of La did not change the crystal structure of the ZnO, and it was uniformly dispersed in the ZnO matrix (Manikandan et al., 2017). The reaction was incomplete when the La doping exceed 6%, resulting in a small amount of Zn(OH)2 remained. Next, unless otherwise specified, mentioned La-ZnO is the sample with a La-doping amount of 4%.

The Fourier transform infrared spectra (FTIR) of the pure and La doped ZnO QDs were displayed in Figure 3B. The intensity band at around 465 cm−1 was attributed to the Zn-O stretching vibration. The band at 605 cm-1 indicates the presence of ZnO and La. The intensity band at 1,635 cm-1 may be contributed to the O-H bending mode of the adsorbed water vibration, and the vibration amplitude of La-ZnO is more obvious than that of pure ZnO, indicating the successful incorporation of lanthanum.

**FIGURE 4:**
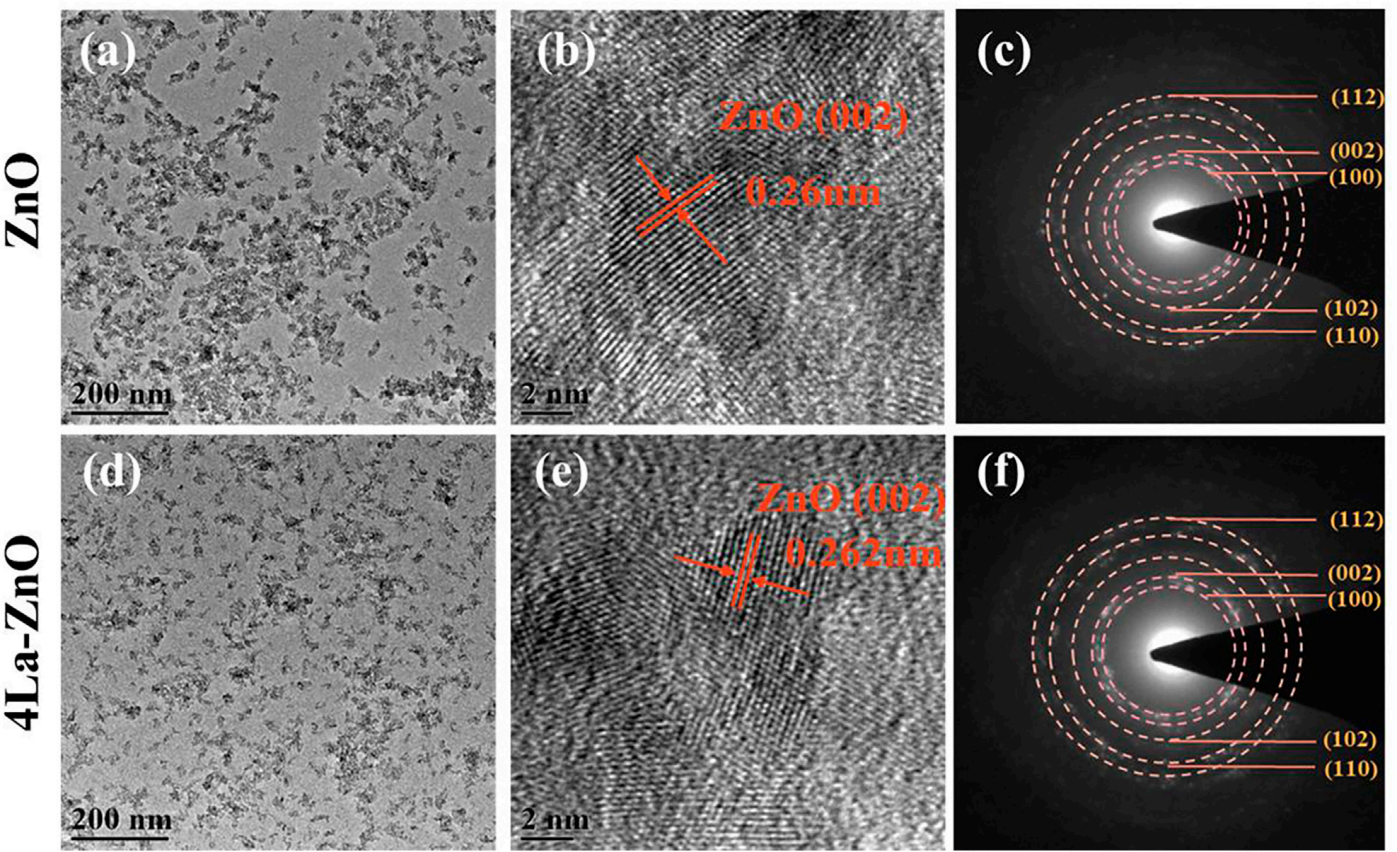
TEM **(A,D)**, HR-TEM **(B,E)** and SEAD **(C,F)** patterns of pure and La-ZnO QDs.

To further investigate the microstructure of La-ZnO QDs, transmission electron microscope (TEM) and selected-area electron diffraction (SAED) measurements were carried out. As shown in Figures 4A,D, both pure ZnO and 4La-ZnO QDs were uniformly dispersed without obvious agglomeration. From the HRTEM images as shown in Figures 4B,E, the pure ZnO exhibited more regular lattice stripes, whereas La-ZnO is less ordered. Besides, the space between the adjacent lattice planes was 0.26 nm, which is consistent with the (002) plane of ZnO (Sun et al., 2012). For La-ZnO QDs, the space lattice plane has increased slightly and reached 0.262 nm, indicating that the doped of rare earth lanthanum causes lattice distortion of ZnO. Selected area electron diffraction patterns (Figures 4C,F) indicate the diffraction ring well corresponds to (112) (002), (100) (102), (110) planes, which is in good agreement with the results of XRD.

**FIGURE 5:**
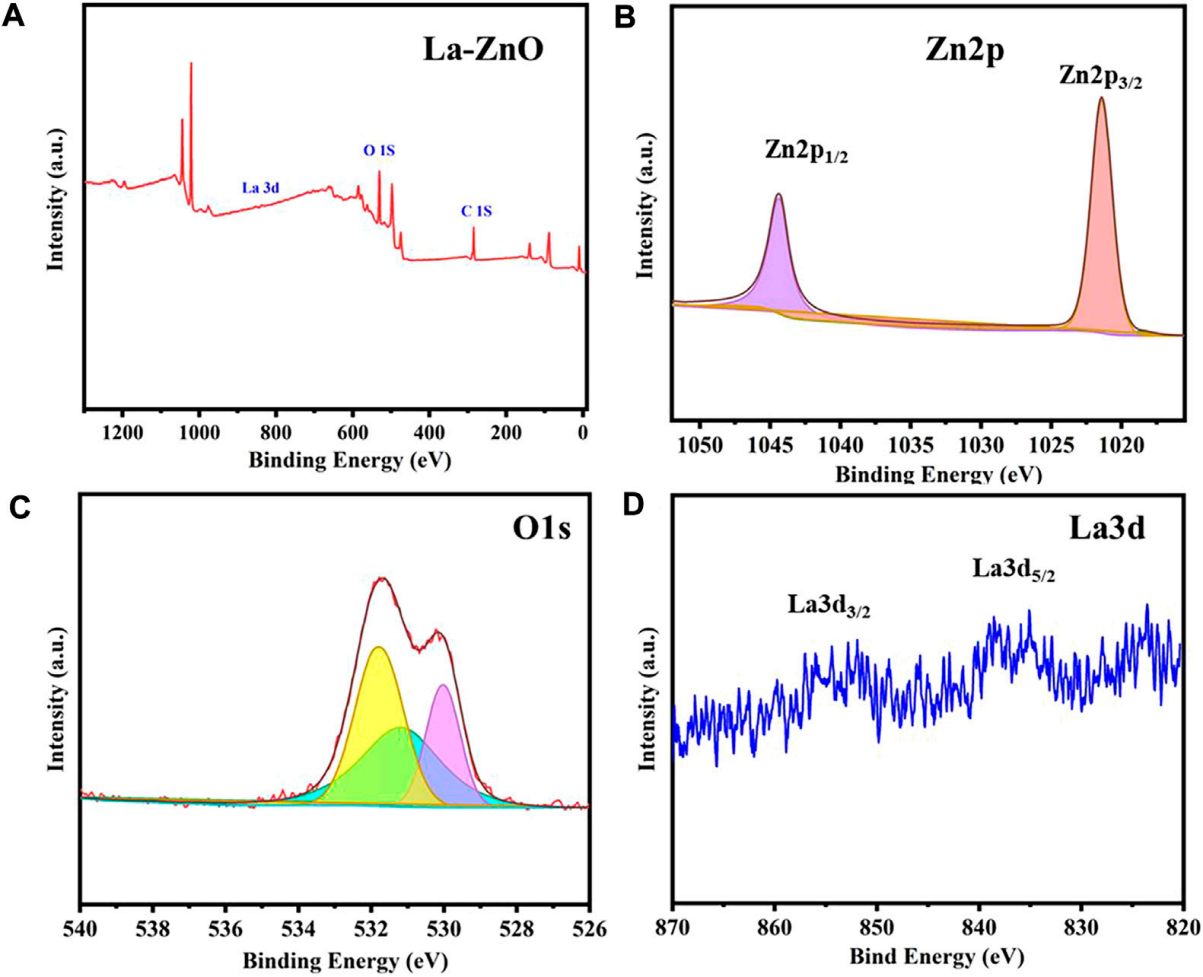
**(A)** XPS survey spectra of La-ZnO. **(B–D)** High-resolution XPS spectra of Zn2p, O1s, and La3d.

The surface element composition and valence of ZnO and La-ZnO QDs were determined by XPS. The binding energy peaks of Zn, La, and O were detected in the La-ZnO composite (Figure 5A), which further confirming the successful doping of lanthanum. Furthermore, high-resolution spectra of Zn2p, O1s, and La3d were provided in Figure 5B, the peaks at 1,023.1 and 1,043.2 eV corresponded to Zn 2p3/2 and Zn 2p1/2, respectively. Figure 5C shows the high resolution of O1 s, the peak at 529.8 eV could be attributed to Zn-O-Zn and the peak at 531.6 eV was attributed to La-O-Zn. It is observed from Figure 5D that the La 3 days spectrum consists of two peaks, the peaks at approximately 836.2 eV and 853.3 eV were ascribed to La3d5/2 and La3d3/2.

**FIGURE 6:**
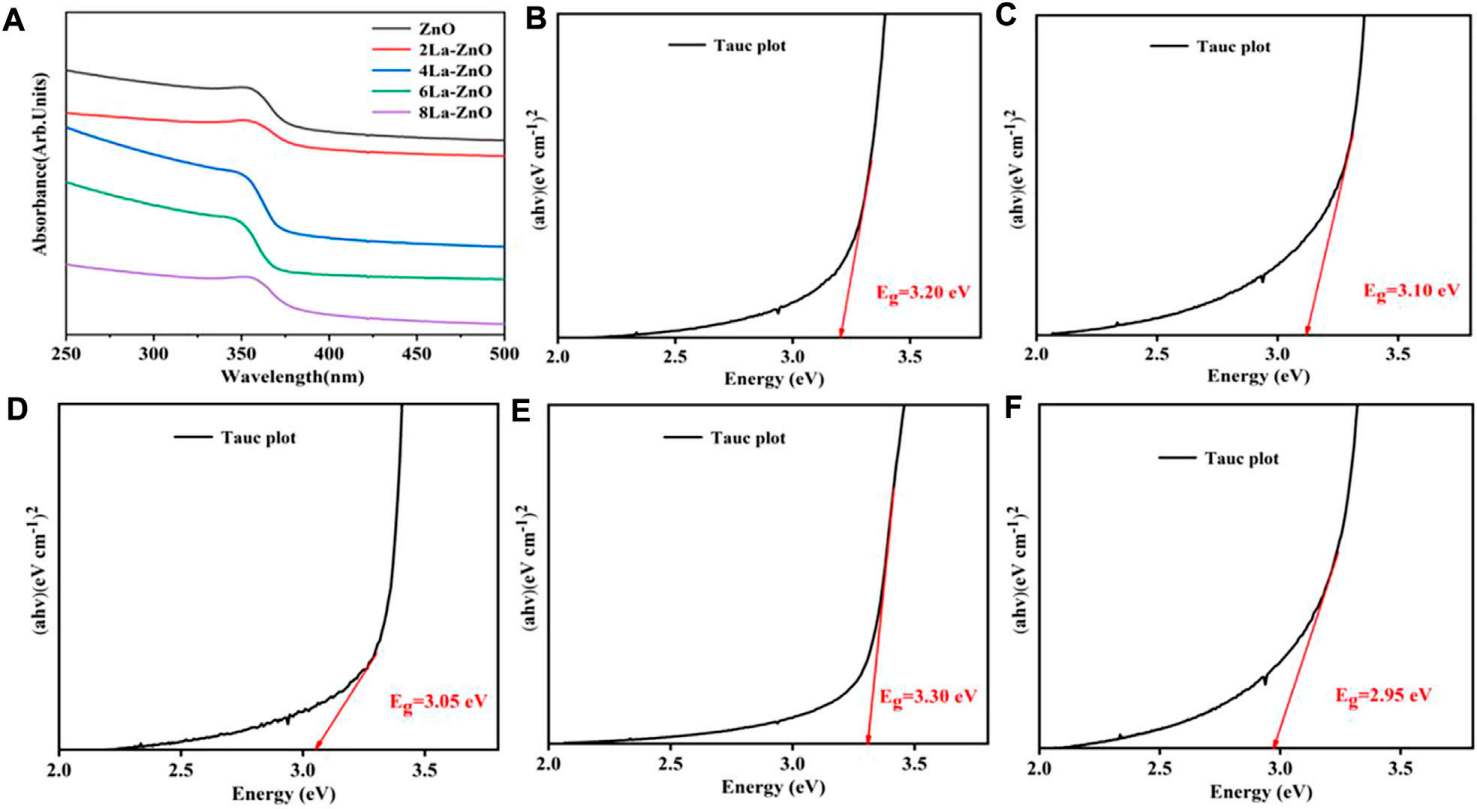
**(A)** UV−vis absorbance spectra of pure ZnO and xLa-ZnO **(B–D)** Tauc plots of (αhν)2 *versus* hν of pure ZnO and xLa-ZnO **(B)** x = 0, **(C)** x = 0.02, **(D)** x = 0.04 and **(E)** x = 0.06, **(F)** x = 0.08.

UV-vis absorption spectroscopy was used to assess the optical properties of pure ZnO and La-ZnO QDs. As shown in Figure 6A, both pure ZnO and La-ZnO QDs exist band edge-absorption at around 366 nm. There is an extended tail band (from 450 to 800 nm) in the UV spectrum of La-ZnO QDs, indicating that they have optical properties almost in the whole visible spectrum. The optical band gap of pure ZnO and xLa-ZnO was calculated by the following formula:

α=A (hυ−Eg)n/ hυ (4)

where *a* is the absorption coefficient, A and n are constant, and *?* is the photon frequency.

Figures 6B–F shows the calculated band gaps of the pure ZnO and xLa-ZnO QDs. The doping of lanthanum has a great effect on the bandgap of xLa-ZnO QDs. When the doping amount of lanthanum increased from 0 to 8%, the bandgap was regularly decreased from 3.35 to 2.85 eV. Thus, doping La into ZnO can introduce impurity levels in the bandgap and enhance its visible light response, when the Zn site in ZnO is occupied by La atoms, two main effects can be observed: 1). The impurity band is close to the edge of the conduction band. 2). Due to the strong orbital coupling between La and O, the obtained band gap decrease (Sun et al., 2012). The results show that the doping concentration of La plays an important role in adjusting the bandgap of La-ZnO, which is conducive to its potential applications in biological imaging.

**FIGURE 7:**
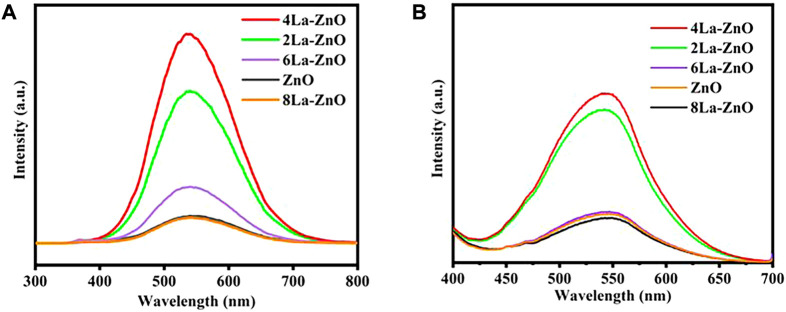
**(A)** PL spectra of pure ZnO QDs and xLa-ZnO QDs. **(B)** Liquid fluorescence spectra of ZnO QDs and xLa-ZnO QDs.

Figure 7A shows the PL spectra of pure ZnO and La-ZnO QDs. From the spectra, it was observed that all emissions of pure ZnO and La-doped ZnO are concentrated around 530 nm. The small variation of the emission position in the PL spectra may be contributed to the effect of the La doping. The maximum emission intensity was increased significantly with appropriate La doping. When the La doping is 4%, the emission intensity reach the maximum. Besides, we also obtained Fluorescence spectra of different sample in distilled water in Figure 7B, and the 4% La-ZnO also showed highest emission intensity. Therefore, in this research, 4% is the best doping amount, and 4La-ZnO QDs are used for the next biological experiment.

### 3.2 Drug loading and release efficiency

HA:CD44, a transmembrane glycoprotein with a molecular weight of 85 kDa, is involved in a variety of cellular functions, including cell orientation, adhesion, migration and stromal cell signaling processes. It is over expressed in many solid tumors. Hyaluronic acid (HA), composed of d-glucuronic acid and n-acetylglucosamine, is a negatively charged linear polysaccharide. It specifically recognizes the CD44 glycoprotein and here HA has been used as a targeting ligand to specifically recognize the CD44 glycoprotein overexpression in murine melanoma cells.

PEG has been widely used in drug delivery as a non-toxic modified ligand due to its excellent properties. It not only has good water solubility, high stability, and good biocompatibility. Therefore, using PEG ligand connection of ZnO quantum dot clusters carrier has many advantages, can not only make use of the EPR effect efficient accumulation in the tumor cells but also can realize drug controlled release. In addition, zinc oxide cluster carrier after complete decomposition, side effects, small decomposition Zn2+ can also be generated and synergistic anticancer, improve the effectiveness of the treatment of tumor.

**FIGURE 8:**
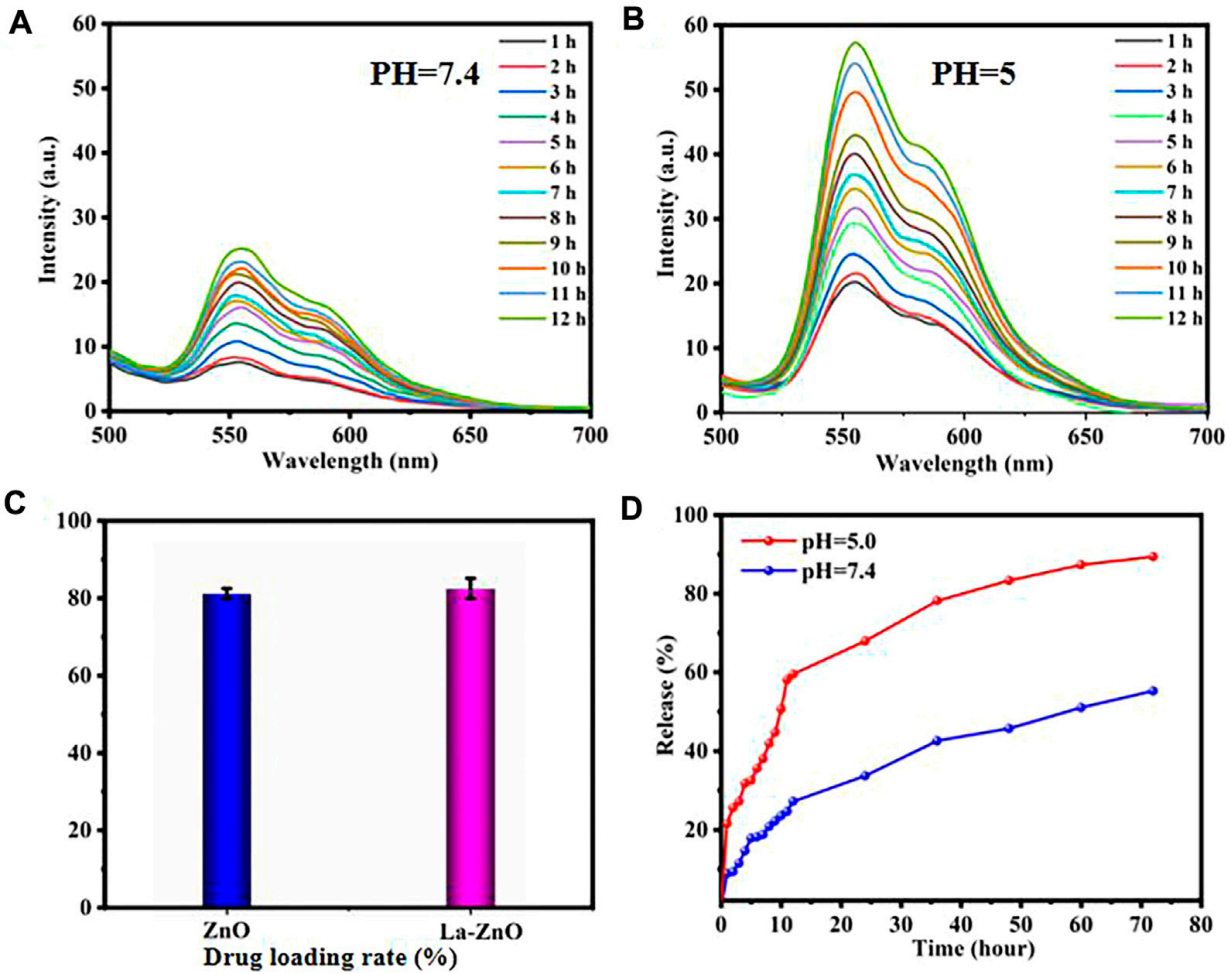
Time-dependent fluorescence spectra of DOX release after incubating La-ZnO-DOX at pH 7.4 **(A)** and pH 5.0 **(B)**.**(C)** Drug loading rate of pure ZnO and La-ZnO.**(D)** Drug release rate of HA-PEG-NH2-Zno-DOX drug loading system at pH = 5.0 and 7.4 with time.

DOX was loaded to the PEG-HA-La-ZnO at a neutral pH of 7.4. DOX can be loaded onto the pure ZnO and La-ZnO by three methods: 1) The complexation of DOX with Zn2+ can provide a payload of DOX molecules. Under acid conditions, the disruption of the coordination bond between Zn2+ and DOX enables the precise release of DOX at the tumor site. 2) DOX can bind to PEG-HA-La-ZnO by an amide bond. 3) Positively charged DOX adsorbed on negatively charged HA-La-ZnO-PEG nanocarriers *via* electrostatic adsorption (Brouet et al., 2008). In this work, DOX loading amounts of PEG-HA-ZnO and PEG-HA-La-ZnO are 81.53% and 81.67%, respectively (Figure 8C).

Generally, TME is acidic and always has a lower pH compared to normal tissues, which is mainly attributed to the production of lactic acid in the anaerobic microenvironment and the formation of hydrolyzed protons during ATP (Adenosine triphosphate) (Vimala et al., 2017; Singh et al., 2018). To improve the therapeutic efficacy of nanoparticles, a large amount of research has been devoted to the fabrication of TME-responsive or cancer-targeted drug delivery platforms. ZnO quantum dots possess pH-responsive release, which exists stably at pH 7.4, but dissolves rapidly at pH < 5.5. To investigate the release of DOX from HA-PEG-La-ZnO quantum dots, PBS buffer solutions (pH = 7.4 and pH = 5) were used to simulate normal cells and tumor microenvironment, respectively. As shown in Figures 8A,B, the fluorescence intensity of DOX at pH = 7.4 is much weaker than in PBS buffer at pH = 5, indicating more DOX was released by ZnO degradation in an acidic environment. In addition, the cumulative release of DOX at different pH was also investigated, as shown in Figure 8D, with the increase of time, DOX was released at a higher rate in pH = 5 solution than in pH = 7 solution. After 72 h of incubation, 70% of DOX was released from the drug-loaded system at pH = 5, while only 32% of DOX was released from the drug-loaded system at pH = 7.4. The above phenomenon is due to the dissolution of ZnO quantum dots and dissociation of drug-metal complexes under acidic conditions, which triggers the release of DOX.

### 3.3 Cytotoxicity assay *in vitro*

**FIGURE 9:**
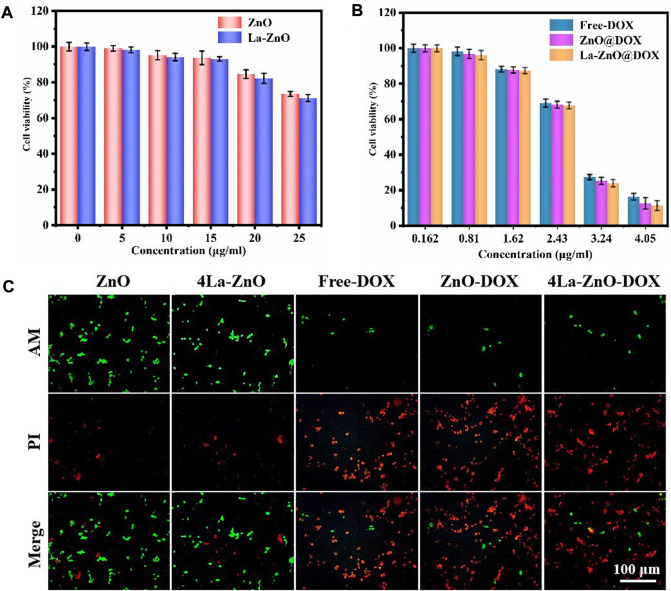
Cytotoxicity study of B16 cells after 48 h of incubation with **(A)** ZnO QDs and La-ZnO QDs. **(B)** Free-DOX, ZnO@DOX and 4La-ZnO@DOX. **(C)** The fluorescent images of the cells treated with different groups and stained with AM/PI.

Drug carriers should not only have high drug-carrying abilities and controlled drug-delivery capability but also possess good biocompatibility (Peppicelli et al., 2017). According to the results of PL, 4% La-ZnO is selected to evaluate the effect of La-ZnO on cellular behaviors, and the concentration-dependent cytotoxicity of pure ZnO QDs and La-ZnO QDs was evaluated with B16 cells. The results, as shown in Figures 9A,B, show that there is no significant toxic effect on both groups at concentrations below 15. Significant antitumor effects were exhibited in both groups when the dose exceeded 25 μg/ml, the survival rate of cytotoxic cells was 78% for the pure ZnO group and 74% for the La-ZnO group, which was attributed to ZnO quantum dots dissolving free Zn2+ into tumor cells. Interestingly, compared to pure ZnO, La-ZnO exhibited a stronger tumor-killing effect. This may be due to the addition of lanthanum causing a decrease in the mitochondrial membrane potential of tumor cells and thus increasing the level of reactive oxygen species (ROS) (He and Shi, 2011). In addition, it is positively correlated with the concentration of DOX particles. The same pattern was observed for the drug loading group, and the toxicity of pure DOX was relatively small, while the ZnO-DOX and 4% La-ZnO-DOX groups showed better cell inhibition. At 2.43 μg/ml of DOX, the cell viability of all the groups was reduced to about 70%, with increasing concentration, the cytotoxicity of all groups decreased significantly, and when the concentration was increased to 4.05, the cellular activity of free DOX, ZnO@DOX and La-ZnO@DOX decreased to 18%, 15%, 12%, respectively. Besides, the La-ZnO@DOX also shows a better anti-tumor effect, which indicates that the synthesized La-doped ZnO quantum dots not only have better fluorescence imaging ability, but also have a better anti-tumor effect, and this result is valuable for follow-up research.

Staining of live and dead cells is also effective way to evaluate the antitumor effect of materials (Shuai et al., 2018). It can be seen from Figure 9C that the cell survival rate of the pure ZnO group is higher, indicating that the low concentration of quantum dots is safe and has no toxic effect on normal cells, while the La-ZnO group presents more dead cells, indicating a better anti-tumor effect, which is consistent with the previous MTT experiment. This indicates that lanthanum doping not only improves the fluorescence effect of ZnO but also shows stronger anti-tumor properties than undoped samples. In addition, when the DOX was loaded, all groups showed large areas of dead cells, indicating that powerful anti-tumor effect of La-ZnO@DOX.

### 3.4 *In vivo* biocompatibility

**FIGURE 10:**
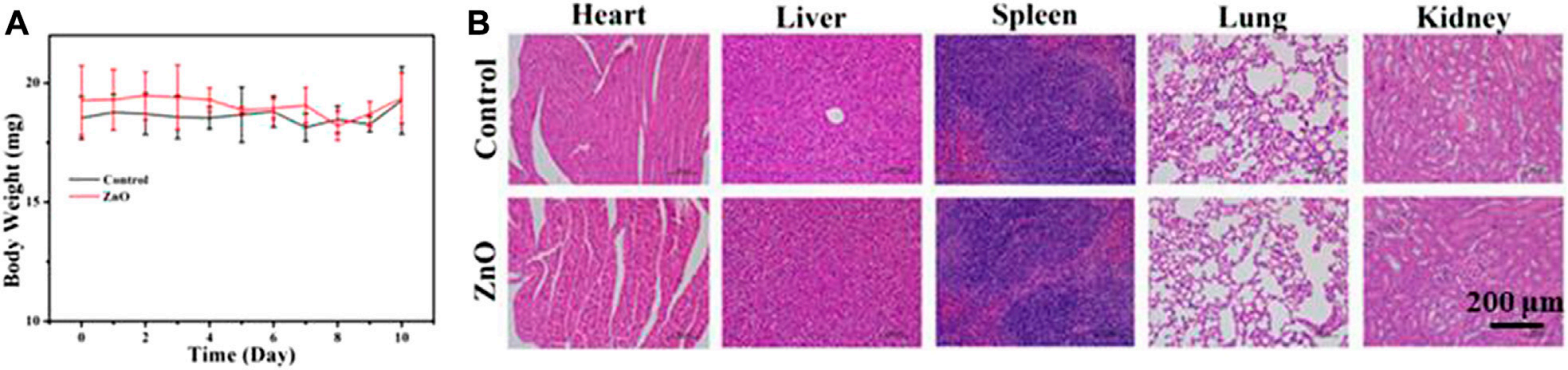
**(A)** The body weight of mice in control group and Zinc oxide group was determined after subcutaneous injection of 100 μL PBS and 25 μg/ml ZnO solution for 10 consecutive days.**(B)** H&E staining images of heart, liver, spleen, lung and kidney after 10 days.

In this study, the B16F10 melanoma model was used to study the biocompatibility of nano-platform *in vivo*, and the potentially toxic side effects of ZnO as a drug carrier on mice were studied. Six normal C57 mice were randomly divided into two groups, which were subcutaneously injected with 100uL PBS and 100 uL 25ug/mL ZnO, respectively. The body weight of mice was recorded for 10 consecutive days, and then the main organs (liver, heart, spleen, lung, and kidney) of mice were collected for H&E staining. The body weight of mice at 10 days was recorded, as shown in Figure 10A. The results showed that there was no significant difference in the body weight of mice between the two groups. Figure 10B shows the staining results of the major organs of mice. The staining results showed that there was no significant difference between the major organs of mice treated with ZnO and the control group. The results show that ZnO had good biocompatibility and had no obvious toxic side effects on normal organs of mice. ZnO could be used as an effective drug carrier for anti-tumor research. The anti-tumor mechanism of lanthanum (La) ions may be attributed to the fact that lanthanum ions open the mitochondrial permeability transition pore, blocking the electron transport chain in the mitochondria, thus increasing the level of reactive oxygen species and inducing cancer cell death (Anghileri et al., 1987; Shuai et al., 2018; Vinothini et al., 2019).

### 3.5 *In vivo* antitumor activity

**FIGURE 11:**
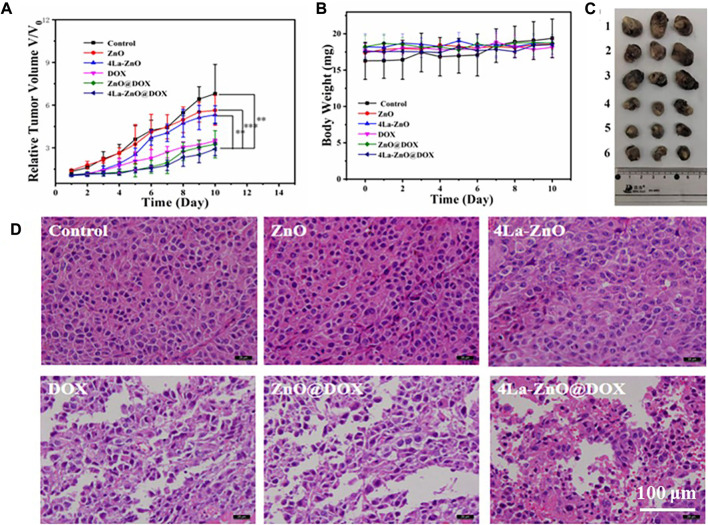
*In vivo* evaluation of antitumor efficacy. **(A)** Mean relative tumor volumes within 10 days.**(B)** The body weight of mice was determined after subcutaneous injection of 100 μL PBS and 25 μg/ml ZnO QDs, La-ZnO QDs, Free-DOX, ZnO@DOX and 4La-ZnO@DOX for 10 consecutive days. **(C)** Representative images of tumors from different groups. Where (1) Control, (2) ZnO, (3) La-ZnO, (4) Free-DOX, (5) ZnO@DOX, (6) 4La-ZnO@DOX. **(D)** Micrographs of H&E stained tumor slices collected from mice of different groups.

In this study, the *in vivo* antitumor efficacy of the nanoplatform in C57 mice was investigated using the B16F10 melanoma model. The tumor-bearing mice were divided into six groups: PBS, DOX, ZnO, 4La-ZnO, ZnO@DOX, and 4La-ZnO@DOX groups. The average relative tumor volume was measured daily. Data were processed by the normalization method. As shown in Figure 11A, ZnO, 4La-ZnO, DOX, ZnO@DOX, and 4La-ZnO@DOX all showed a certain degree of tumor inhibition compared with the control group, among which 4La-ZnO@DOX showed a stronger tumor growth inhibition effect, which was consistent with the results of cell experiments and staining experiments. Figure 11B shows the weight change of mice. Within 10 days after treatment, there is no significant difference in the weight of each group, which further verifies that ZnO has no obvious side effects and does not cause other toxic damage to the normal organs of mice, and has good biocompatibility. In addition, Figure 11C shows the size of the mouse tumor. The mean relative tumor volume in the 4La-ZnO@DOX group was smaller than that in the DOX and ZnO@DOX groups, indicating that the antitumor effect of 4La-ZnO@DOX was improved. The tumors of each mouse were collected for HE staining. Figure 11D shows the staining results of tumor sections. Compared with the control group, the tumor sections of the treatment group showed larger areas of necrosis and apoptosis, showing a better tumor inhibition effect. ZnO has the advantages of PH-responsive degradation, good dispersion and biocompatibility, so that 4La-ZnO@DOX has high tumor suppression efficiency. ZnO has the advantages of PH-responsive release and is selectively released in the tumor microenvironment. When the ZnO drug loading system enters the mice and accumulates in the tumor site, ZnO decomposes Zn2 +, which has certain cytotoxicity and can kill cancer cells. La3+ ions can induce endoplasmic reticulum-dependent apoptosis through Bcl-2/Bax cascade, leading to apoptosis of B16 cells (Yu et al., 2015). demonstrated that the antitumor mechanism of La3+ was partly attributed to miRNAs let-7a and Mir-34a (Wu et al., 2013). found that lanthanum induced primary neuronal apoptosis through mitochondrial dysfunction regulated by Ca2 and Bcl-2 families. Therefore, a lanthanum-doped zinc oxide drug delivery system could achieve a stronger antitumor effect.

## 4 Conclusion

Multifunctional lanthanum-doped zinc oxide (La-ZnO) quantum dots were successfully synthesized. The PEG-HA-La-ZnO QDs has the best fluorescence properties when the doping amount is 4%. PEG-HA-La-ZnO QDs exhibited better anti-tumor effect undoped samples. Dual enhancement of fluorescence and anti-tumor effect by La doping makes La-ZnO QDs promising in drug delivery system applications.

## Data Availability

The raw data supporting the conclusions of this article will be made available by the authors, without undue reservation.
